# Supplementary material for: The Evolution and Characterization of the RNA Interference Pathways in Lophotrochozoa
Source: Genome Biol Evol. 2024 May 7;16(5):evae098. doi: 10.1093/gbe/evae098 (PMC11114477; doi:10.1093/gbe/evae098)
Supplement: evae098_Supplementary_Data [file evae098_supplementary_data.zip › Supplementary_materials.zip!/selected_miRNAs/CrGi_selected_miRNAs/CrGi_NC_047559.1_2455.pdf]

Provisional ID	:	NC_047559.1_2455
Score total	:	27.4
Score for star read(s)	:	3.9
Score for read counts	:	21.5
Score for mfe	:	0.9
Score for randfold	:	1.6
Score for cons. seed	:	-0.6
Total read count	:	54
Mature read count	:	44
Loop read count	:	0
Star read count	:	10

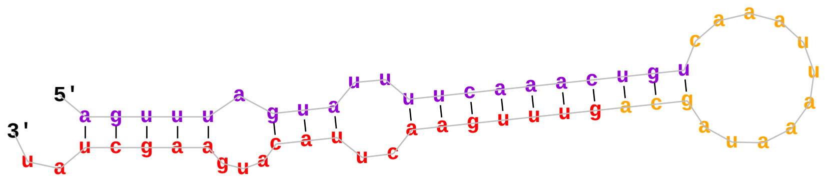

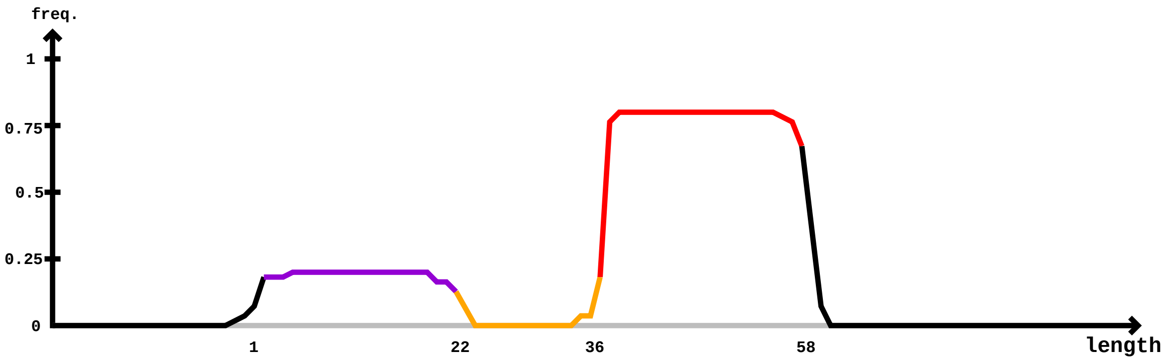

Star Mature

```
5'- acuaugacuauuauuagaauaguuuaguauuuucaaacugucaaauuaaaauagcaguuugaacuuacaugaagcuauuuugaguggucauacuuucaccagugauugccug  -3'  obs
    acuaugacuauuauuagaauaguuuaguauuuucaaacugucaaauuaaaauagcaguuugaacuuacaugaagcuauuuugaguggucauacuuucaccagugauugccug       exp
    ..(((((((((..((((((((((((.(((..((((((((((............))))))))))..)))...)))))))))))))))))))))....................   reads  mm  sample
    ...................uaguuuaguauuuucaaacug........................................................................   1      0   Ov1
    ....................aguuuaguauuuucaaacugu.......................................................................   3      0   Ov1
    ........................................................guuugaacuuacaugaagcua...................................   1      0   Ov1
    .........................................................uuugaacuuacaugaagcua...................................   1      0   Ov1
    .........................................................uuugaacuuacaugaagcuau..................................   3      0   Ov1
    .........................................................uuugaacuuacaugaagcuauu.................................   3      0   Ov1
    .........................................................uuugaacuuacaugaagcuauuu................................   2      0   Ov1

    .................aauaguuuaguauuuucaaac..........................................................................   1      0   Ov2
    ....................aguuuaguauuuucaaacugu.......................................................................   1      0   Ov2
    .......................uuaguauuuucaaacugu.......................................................................   1      0   Ov2
    .........................................................uuugaacuuacaugaagcuau..................................   3      0   Ov2
    .........................................................uuugaacuuacaugaagcuauu.................................   1      0   Ov2
    .........................................................uuugaacuuacaugaagcuauuu................................   1      0   Ov2

    ..................auaguuuaguauuuucaaac..........................................................................   1      0   Mt2
    ...................uaguuuaguauuuucaaacug........................................................................   1      0   Mt2
    ....................aguuuaguauuuucaaacugu.......................................................................   1      0   Mt2
    ......................................................caguuugaacuuacaugaagcua...................................   1      0   Mt2
    ........................................................guuugaacuuacaugaagcuau..................................   5      0   Mt2
    .........................................................uuugaacuuacaugaagcu....................................   1      0   Mt2
    .........................................................uuugaacuuacaugaagcuau..................................   2      0   Mt2
    .........................................................uuugaacuuacaugaagcuauu.................................   4      0   Mt2
    .........................................................uuugaacuuacauUaagcuau..................................   1      1   Mt2
    .........................................................uuugaacuuacaugaagcuauuu................................   1      0   Mt2
    ..........................................................uugaacuuacaugaagcuau..................................   1      0   Mt2

    ....................aguuuaguauuuucaaacugu.......................................................................   1      0   Mt1
    ......................................................caguuugaacuuacaugaagcua...................................   1      0   Mt1
    ........................................................guuugaacuuacaugaagcuau..................................   2      0   Mt1
    .........................................................uuugaacuuacaugaagc.....................................   1      0   Mt1
    .........................................................uuugaacuuacaugaagcuau..................................   4      0   Mt1
    .........................................................uuugaacuuacaugaagcuauu.................................   4      0   Mt1
```

Star Mature

```
acuaugacuauuauuagaauaguuuaguauuuucaaacugucaaauuaaauagcaguuugaacuuacaugaagcuauuuugaguggucauacuuucaccagugauugccug
.........................................................uugUacuuacaugaagcua....................................     1     1     Mt1
```
